# Differences in Person-Centered Care in Fetal Care Centers: Results from the U.S. Pilot Study of the PCC-FCC Scale

**DOI:** 10.3390/jpm14070772

**Published:** 2024-07-20

**Authors:** Abigail B. Wilpers, Katie Francis, Amy B. Powne, Lonnie Somers, Yunyi Ren, Katherine Kohari, Scott A. Lorch

**Affiliations:** 1 Department of Family and Community Health, University of Pennsylvania School of Nursing, Philadelphia, PA 19104, USA; 2 Research Institute, Children’s Hospital of Philadelphia, Philadelphia, PA 19104, USA; 3 Fetal Therapy Nurse Network, Chicago, IL 60604, USA; 4 North American Fetal Therapy Network, Roseville, MN 55113, USA; 5 St. Louis Fetal Care Institute, SSM Health Cardinal Glennon Children’s Hospital, St. Louis, MO 63104, USA; 6 UC Davis Fetal Care and Treatment Center, UC Davis Health, Sacramento, CA 95817, USA; 7 Fetal Health Foundation, Littleton, CO 80127, USA; 8 Department of Public Health Sciences, School of Medicine, University of California-Davis, Sacramento, CA 95616, USA; 9 Department of Obstetrics, Gynecology & Reproductive Sciences, Yale School of Medicine, New Haven, CT 06510, USA; 10 Fetal Care Center, Yale New Haven Hospital, New Haven, CT 06510, USA; 11 Division of Neonatology, Children’s Hospital of Philadelphia, Philadelphia, PA 19104, USA; 12 Perelman School of Medicine, University of Pennsylvania, Philadelphia, PA 19104, USA

**Keywords:** nursing research, maternal–fetal medicine, person-centered care, fetal care center, prenatal diagnosis, fetal anomalies, fetal therapy

## Abstract

Objective: We report findings from a U.S. mixed-methods pilot study of the Person-Centered Care in Fetal Care Centers (PCC-FCC) Scale. Methods: Participants, who received care at a U.S. Fetal Care Center (FCC) between 2017 and 2021, completed an online questionnaire providing sociodemographic details, specifics about the care received, qualitative experiences, and scores from the PCC-FCC Scale. Results: Participants’ (*n* = 247) PCC-FCC scores and qualitative feedback indicate high perceived person-centered care (PCC), particularly in areas of care coordination, respectful care, and patient education. However, 8% scored below the midpoint, and 38% of comments were negative, especially regarding expectation setting, preparation for post-intervention maternal health, and psychosocial support. Public insurance was associated with higher total PCC-FCC (*p* = 0.03) and Factor 2 scores (*p* = 0.02) compared to those with private insurance. The qualitative themes trust, clarity, comprehensive care, compassion, and belonging further elucidate the concept of PCC in FCCs. Conclusion: The PCC-FCC Scale pilot study revealed strong overall PCC in FCCs, yet variability in patient experiences suggests areas needing improvement, including expectation setting, preparation for post-intervention maternal health, and psychosocial support. Future research must prioritize diverse samples and continued mixed methodologies to better understand the role of insurance and identify other potential disparities, ensuring comprehensive representation of the FCC patient population.

## 1. Introduction

Person-centered care (PCC) has gained widespread recognition as fundamental to quality healthcare delivery, extending into high-risk perinatal care contexts, where its significance is increasingly emphasized [1,2,3]. PCC prioritizes the individual’s preferences, needs, and values, fostering a collaborative and respectful relationship between patients and healthcare professionals [4]. Specialized perinatal settings like fetal care centers (FCCs) play a crucial role in providing PCC to individuals facing complex prenatal diagnoses of fetal conditions such as congenital heart disease (CHD) and spina bifida. These patients rely on FCCs not just for advanced interdisciplinary medical care but also for emotional support, coping strategies for the loss of a normal pregnancy, and assistance in navigating complex decision-making processes, including determining the course of a pregnancy amidst life-threatening or life-limiting fetal conditions [5,6,7]. However, evidence to support PCC in these unique care settings has been lacking. This was the impetus to develop an instrument capable of assessing the quality of PCC within FCCs [1].

The development of the Person-Centered Care in Fetal Care Centers (PCC-FCC) Scale engaged both FCC clinicians and patient representatives across the U.S. This community-engaged approach supported diverse perspectives on indicators of PCC, encompassing aspects such as respectful communication, emotional support, involvement in decision-making, and coordination of care. The recent pilot study of the PCC-FCC Scale demonstrated its reliability and validity for assessing PCC within U.S. FCCs [1]. While this analysis provided valuable insights into the psychometric properties of the PCC-FCC Scale and advanced the development of standardized measures, it did not explore associations between patient factors, such as socioeconomic status (SES), and their FCC experiences. It is essential to examine the intricate contextual variations in PCC experiences—an exploration that benefits from a mixed-methods approach.

Our objective was to explore both quantitative and qualitative assessments of pilot participants’ experiences in FCCs to identify common patterns and potential sociodemographic disparities. This approach ensures that significant nuances are thoroughly examined before implementing standardized measures [8,9]. The goal of the continued development of the PCC-FCC Scale is to improve care quality for individuals and families dealing with complex prenatal diagnoses.

## 2. Materials and Methods

We used a convergent parallel mixed-methods design in which quantitative and qualitative data were collected concurrently, analyzed independently, and then merged [10]. The combined results were analyzed using a joint display to establish convergence.

### 2.1. Sample

We employed convenience sampling to recruit individuals who had received care at an FCC in the U.S. between 2017 and 2021, were aged 18 or older, had Internet access, and were proficient in English. The inclusion criteria were intentionally broad to ensure a large sample size.

Recruitment notices targeting former FCC patients were disseminated across various online platforms, including Internet forums, blogs, informational websites, social media platforms, and online support communities relevant to fetal diagnosis and treatment (e.g., the Fetal Health Foundation, the Spina Bifida Association). Information about the study was also distributed through the North American Fetal Therapy Network and Fetal Therapy Nurse Network, allowing members to promote the study on their platforms or engage in targeted recruitment efforts. A total of eight sites contributed to targeted recruitment efforts (UPMC Magee-Women’s Hospital; Riley Children’s; Miller Children’s and Women’s Hospital Long Beach; University of California, Davis; Children’s Minnesota; Children’s Mercy Kansas City; Yale New Haven Health; and Children’s Wisconsin). Targeted recruitment involved screening electronic medical records to identify eligible former patients, who were subsequently notified about the study via email, phone call, letter, or a patient–provider messaging platform. Institutional Review Board approval was obtained from [blinded for peer review], and participating sites either granted approval or were exempt from review (protocol # 2000032383). Participants did not receive any compensation for their involvement.

### 2.2. Data Collection

Cross-sectional data were collected from each participant using a secure web-based self-administered survey hosted on Qualtrics. The study details were presented to potential participants on the survey’s initial page. A participant screening tool was utilized to assess eligibility, which included a comprehensive list of sites meeting the defined criteria for an FCC [1].

Upon confirming eligibility, written informed consent was obtained, followed by completion of a sociodemographic questionnaire, which included items on financial challenges and perceptions of discrimination (Table 1). Participants then completed the 28-item, 2-factor PCC-FCC Scale [1]. Responses to PCC-FCC items were recorded using a Likert scale ranging from 1 (disagree strongly) to 4 (agree strongly), with an additional option for “does not apply”. PCC-FCC scores range from 28 to 112, with higher scores indicating a higher degree of perceived PCC [1]. Factor 1 scores, ranging from 19 to 76, reflect the sense of being cared for through respectful and compassionate caregiving tasks, such as technical competence and effective communication. Factor 2 scores, ranging from 9 to 36, signify feeling cared about by addressing health-related quality-of-life needs, including genuine provider connections and meeting psychosocial needs. The development and validation of this scale is described in detail by Wilpers et al. (2024) [1].

We initially utilized the Revised Patient Perception of Patient-Centeredness (PPPC-R) Questionnaire, an 18-item validated instrument designed to measure patients’ perceptions of patient-centered care [11], to assess concurrent validity [1]. We found a highly significant correlation between the total scores of the PPPC-R and the Patient-Centered Care-Family Caregiver (PCC-FCC) Scale (r = 0.889, *p* < 0.001). In this pilot study, we continued to use the PPPC-R as a validated benchmark for interpreting the results of the pilot PCC-FCC study. This approach allowed us to identify potential differences and progressively deepen our comprehension of the novel PCC-FCC Scale.

Qualitative data were collected from an open-ended prompt at the end of the survey: “Is there any other information about your care experience that you feel is important to share to help us measure and enhance care quality?”

### 2.3. Data Analysis

#### 2.3.1. Quantitative Analysis

For each questionnaire (PCC-FCC and PPPC-R), participants with less than 70% items of qualified survey responses were excluded from the analysis. The mean PCC-FCC and PPPC-R scores were stratified by sociodemographic factors, with sufficient representation across groups (Table 1). We limited our analysis to variables with at least 10 participants in each group to ensure reliable results. This threshold enhances validity and avoids biases from small sample sizes. Wilcoxon or Kruskal–Wallis rank-sum tests were used as needed for testing differences. The changes in scores between pre-COVID-19 and post-COVID-19 were also examined. All statistical analyses were performed using R version 4.1.3 within RStudio.

#### 2.3.2. Qualitative Analysis

We analyzed the qualitative data to identify distinctive themes of PCC in FCCs and identify descriptions of potential disparities. We also sought to uncover any concepts that might be absent from or require further development in the current PCC-FCC Scale items. Thematic and content analyses were employed to interpret participants’ responses to the open-ended question, where they recounted their individual FCC experiences [12]. Qualitative data analysis was conducted using Atlas.ti 8 software (Berlin, Germany). In vivo, descriptive, and conceptual codes were employed by A.W. and K.F. to create the initial coding framework based on the participants’ narrative content. This process involved ongoing consultation and review with the entire research team to ensure comprehensiveness. A codebook was established, and coding comparisons continued until consensus was reached between coders. These codes were then organized into themes to capture the components of PCC in FCCs for this sample. Analytic memos were generated, and the software’s concept-mapping feature facilitated the identification of connections across codes and the dataset. Additionally, an audit trail was regularly updated after each research team meeting and data analysis session to ensure methodological rigor.

### 2.4. Integration

The integration of qualitative and quantitative data in this study followed established principles of mixed-methods research. We began with data triangulation, where findings from both methods were compared and contrasted to identify common patterns and discrepancies, ensuring that the data were examined from multiple perspectives to enhance their validity and reliability. This was followed by a joint display analysis, which visually mapped the quantitative results alongside qualitative themes. This approach facilitated a deeper interpretation by allowing for the simultaneous examination of numerical and thematic findings, highlighting areas of convergence and divergence. The final step involved narrative weaving, where quantitative results were embedded within the qualitative findings section to illustrate and enrich the numerical findings. This comprehensive and structured approach ensures rigorous and nuanced integration of data, providing robust and meaningful findings consistent with recognized standards in mixed-methods research.

## 3. Results

### 3.1. Sample

A total of 247 participants, representing most U.S. regions with FCCs, completed over 70% of the PCC-FCC Scale between January 2022 and May 2023, meeting the eligibility criteria for inclusion in the analysis. The sociodemographic and care characteristics of the sample and the excluded participants are summarized in Table 1. The participants were primarily White (89.9%), married (86.8%), held private insurance (86.8%), and received care in the Midwest (60.9%). Attrition analysis indicated that the final sample did not significantly differ from the excluded participants (*n* = 11) in these respects. However, a significant difference was observed in their total scores, which was anticipated, as each answered item contributes to the overall score. More heterogeneity was seen in the types of care that the participants had received, which were reported as different combinations of consultations, expectant management, fetal intervention, neonatal intervention, palliative care, and termination of pregnancy. A small proportion of participants (5.7%) reported feeling discriminated against in their care. However, this subgroup spanned diverse sociodemographic backgrounds, with no discernible patterns except for the notable inclusion of two out of the five participants who did not identify with the gender pronouns she/her/hers.

### 3.2. Quantitative Findings

A total of 247 participants completed >70% of the PCC-FCC survey (*N* = 237 for PPPC-R survey) and were included in the quantitative analysis (Table 2). The PCC-FCC (median [IQR] = 102 [89, 108]) and PPPC-R scores (3.8 [3.6, 4.0]) showed high perceived PCC overall. However, 8% of participants scored below the midpoint for PCC-FCC total scores. The Factor 1 scores ranged from 26 to 76, with an average of 68.21 (SD = 20.29), while the Factor 2 scores ranged from 9 to 36, with an average of 28.52 (SD = 6.49). A summary of item scoring from highest to lowest mean is presented in Table 2, with the lower items often showing the greatest variability in responses. Notably, the items with the lowest scores and highest variability often stemmed from Factor 2 and pertained to the psychosocial and follow-up aspects of care.

We found no significant differences in PCC-FCC or PPPC-R scores among the years (*p* = 0.632; *p* = 0.749), nor were there discernible differences between care received pre- and post-COVID-19 (*p* = 0.664; 0.849). Table 3 shows the associations between PCC-FCC and PPPC-R scores by sociodemographic groups. Again, this analysis was limited to household income, insurance status, and education level, due to insufficient representation across gender, race, ethnicity, relationship status, employment status, and primary language (Table 1). Participants with public insurance had higher total PCC-FCC scores (*p* = 0.011) and Factor 2 scores (*p* = 0.008) than those with private insurance (Table 3). These associations were not significant for PPPC-R scores. No other significant associations were found between participant characteristics and PCC-FCC scores (Table 3).

### 3.3. Qualitative Findings

Of the 247 participants included in our analysis, 71 (29%) provided qualitative data. The characteristics of the qualitative cohort did not significantly differ from those of the larger sample. Sentiment analysis within the 71 comments revealed a spectrum of responses that broadly corresponded with the breakdown of the quantitative findings. The majority of cases (54%) described a positive PCC experience, while fewer participants (38%) described negative experiences. Sentiment analysis was further stratified according to the five overarching themes of PCC in FCCs that we identified: (1) trust, (2) comprehensive care, (3) compassion, (4) clarity, and (5) belonging. Notably, we did not observe any sociodemographic pattern in the distribution of positive versus negative comments. However, important details related to social determinants of health emerged in several comments. These details are included in Table 4 and below to further elucidate their clinical relevance.

Trust: Defined as a patient’s perception of the FCC team’s credibility and reliability, trust was identified as a central theme. While some comments referred to traditional indicators such as credentials and experience, trust was predominantly linked to the team’s ability to provide PCC amid uncertainty. Participants stressed the importance of collaborative planning rooted in the realities of fetal care uncertainties, encompassing diagnostic ambiguity and experimental interventions. Instances where uncertainties were not identified or clearly communicated left patients feeling surprised.

Our doctor gave us false hope and we made plans based on that info (said he didn’t think she had a coarctation, but she did, it was very severe). We didn’t book a postnatal doula because we didn’t anticipate being separated. Participant 99; 33, White, Midwest, private insurance, employed full-time, married, 50K–74K, master’s degree; received expectant management in 2020.

Trust was established when FCC teams demonstrated honesty and empathy in addressing uncertainty, providing patients information about FCCs that might offer a second opinion or greater expertise in managing particular conditions. In these complicated and unpredictable healthcare settings, patients often found it difficult to understand when they needed to advocate for themselves. Instead, they trusted their care team to advocate for them, emphasizing the need for the team to have a deep and evolving understanding of their individual preferences, needs, and values. Unfortunately, this trust was not always met with adequate support and clear communication.

Experiences of discrimination and a lack of transparency from clinicians shaped some participants’ perceptions of trust within FCCs. These narratives highlighted specific sociodemographic factors, sometimes in unexpected ways. For example, a 30-year-old, affluent, and college-educated White woman who received care in the Midwest expressed surprise at not being offered abortion as an option, stating “I was not given the option for abortion which I thought was strange. I believe it had to do with my economic/cultural/race, etc., status” (Participant #84). Despite this experience, she did not report feeling discriminated against in her care (Table 1). This observation challenges the assumption that discrimination within FCCs would exclusively affect individuals from marginalized communities, underscoring the complexity of discriminatory experiences. In addition to this participant, 16 others reported that they were not informed of all of the options for their pregnancy (Table 2, item mean: 3.60), spanning diverse sociodemographic backgrounds with no discernible patterns.

Participants with positive comments about trust (*n* = 7) had a mean total PCC-FCC score of 103 (SD = 7), compared to those with negative comments *(n* = 15), whose mean total score was 75 (SD = 20) (Table 4).

Clarity: We defined clarity as patients understanding the information, instructions, and healthcare services important for the health of both themselves and their fetus/baby. Participants highlighted clarity alongside compassion in person-centered communication. They emphasized the importance of a clear consensus from the entire FCC team regarding diagnosis, prognosis, planning, and anticipated outcomes for both the fetus/baby and the pregnant individual. Focusing solely on clear expectations for the fetus/baby posed challenges, as noted:

I did not fully understand what I was signing up to happen to my body post [maternal-fetal] surgery. I was only thinking about the baby, and it was never fully explained to me. It wouldn’t have changed by decision, but I might have been more prepared to face a lot of difficulty. Participant 174: 31, White, Midwest, private insurance, homemaker, bachelor’s degree, married, 75K–99K; received fetal intervention in 2018.

While this participant mentioned that their decision-making remained unaffected by the lack of clarity, others did express that additional clarity might have influenced their care choices. One participant said “I wish they would have given more time and discussed my options. I wish they would have made it more clear that my baby’s condition would result in death” (Participant 72: 34, White, Midwest, private insurance, employed full-time, bachelor’s degree, married, >100K; received perinatal palliative care in 2019). Another participant described her experience of realizing a lack of clarity regarding what should prompt her to go to the hospital and what to prioritize in her decision-making:

I wish more emphasis would have been placed on kick counts and my feelings as a pregnant mother and less on how many weeks gestation. It is difficult to balance the baby’s development against the risks. If I had gone to the hospital sooner, instead of waiting because so much emphasis had been placed on getting to 38 weeks, it may have changed the outcome for our son. Participant 81; 32, White, Midwest, private insurance, employed full time, bachelor’s degree, married, 75K–99K; received expectant management in 2018.

This quote underscores the emotional toll of unclear communication on pregnant individuals, highlighting the need for a clear understanding of complexities, which can affect decision-making and outcomes.

Despite the majority of qualitative comments related to clarity being negative (Table 4), it is worth noting that the two highest-scoring items on the PCC-FCC Scale were also related to clarity (Table 2). Participants with positive comments about clarity (*n* = 6) had a mean total PCC-FCC score of 106 (SD = 5), compared to those with negative comments (*n* = 17), whose mean total score was 80 (SD = 18) (Table 4).

Comprehensive care: This theme was characterized by patients feeling able to access well-coordinated services that focus on both them and their fetus/baby, guiding and supporting them through transitions throughout their care journey. At certain locations, this involved providing social support services, including access to low-cost temporary housing near the hospital. This was particularly important considering that 25% of participants reported experiencing financial challenges related to their care (Table 1).

Care coordination, a defining element of FCCs, was often highly scored (Table 2).

The [nurse] coordinator who helped me schedule my appointments and kept everything coordinated for me was sooo helpful. She was always available to help me and listened to me and the needs of our family… it felt like we had a personal coordinator. Definitely the best part of the experience knowing I was being cared for and that lessened the stress on me. Participant 141: 27, White, Midwest, private insurance, homemaker, some college, married, 20K–34K; received expectant management in 2019.

Although several participants praised their FCC’s effective “wrap-around service”, unmatched “care continuity”, and excellent communication and coordination with their local providers, others expressed feeling abandoned by the team after childbirth, especially in regard to their own health and wellbeing.

After I gave birth, I was forgotten about. No one made sure that I had a 6-week postpartum checkup scheduled for back home. No one made sure that some type of therapy was set up for me. It was all about my son (he was completely healthy) just no one did anything to my benefit… someone should have made sure I was taken care of before I went home. Participant 176: 22, Black or African American, Northeast, private insurance, homemaker, high school graduate, diploma or the equivalent, married, 20K–34K; received fetal intervention in 2020.

This gap in care represents both a barrier to care continuity and a lack of comprehensive caring for the pregnant person as well as their fetus/baby. Participants often found themselves prioritizing the health of their fetus/baby as mothers, yet they expressed a desire for their FCC teams to advocate for their own health and needs.

I reflect often that I was very much in a mode of thinking of only my unborn children that I did not take great care of myself emotionally/mentally and physically. [Mental health] resources would have been more helpful earlier on since I was already in an overly stressed mental state when that occurred and may have helped with the grieving process. Participant 68: 27, White, Midwest, private insurance, employed full-time, master’s degree, married, >100K; received perinatal palliative care in 2018.

This discrepancy underscores the overshadowing of the pregnant person’s health by the emphasis on the fetus or baby’s wellbeing. This was particularly evident in the emotional and psychosocial health needs of those undergoing maternal–fetal surgery.

I think there should be more mental and physical care options given to moms after baby is born. Physical therapy and speaking with a therapist should have been recommended and set up. That was not the case, and I definitely needed it. Participant 188: 27, White, Midwest, public insurance, homemaker, some college, married, 35K–49K; received fetal intervention in 2018.

Without comprehensive care from their FCCs, patients faced complications for their own health.

Follow ups with mothers is important. I have spoken with many fetal surgery mothers who had several complications after birth that none of us were prepared for or even understood. It was hard to dx and get treatment because some professionals don’t understand the surgery itself or the extent. Participant 215: 30, White, Midwest, private insurance, employed full-time, some college, 50K–74K; received fetal intervention in 2020.

Participants with positive comments about comprehensive care (*n* = 11) had a mean total PCC-FCC score of 107 (SD = 5), compared to those with negative comments (*n* = 16), whose mean total score was 83 (SD = 21) (Table 4).

Compassion: We defined this theme as patients feeling understood, empathized with, and cared about. All participants emphasized the emotional challenges of navigating a pregnancy complicated by fetal conditions, with one calling it “the most stressful time of my life” and another describing her diagnosed post-traumatic stress disorder (PTSD). They deeply valued FCC clinicians who showed genuine compassion by listening, showing their emotions, being kind and nurturing, and sending sympathy cards when a loss was experienced. One noted “Every team member went above and beyond to ensure myself AND my baby were very well cared for with compassion, close monitoring, and empathy with every visit” (Participant 66: 27, White, Midwest; private insurance, employed full time, some college, 75K–99K; received neonatal intervention in 2018).

Communication about severe conditions and poor prognoses greatly influenced perceived compassion. Some participants were dissatisfied with how information was provided, like one who explained “My babies’ condition was fatal, and I was only told how serious it was once in a rushed meeting” (Participant 185: 29, White, Southwest, private insurance, employed full-time, master’s degree, married, >100K; received fetal intervention in 2020). Additionally, a subtheme showed the challenge of presenting a balanced perspective, addressing concerning aspects of a condition, such as Down syndrome, alongside potential positives, like a well-functioning heart. To show compassion, FCC clinicians also had to adapt to patients’ emotional states, recognizing individual variability in reactions to diagnoses.

My husband and I came into the appointment positive, excited, joyful, and left feeling defeated, manipulated and just angry. NOTHING positive was spoken about my baby’s possible diagnosis, as if it was a horrible thing. I did go back to the office when my son was 4 months old and gave my feedback. Hopefully they were able to make some changes in that office on how they give a prenatal diagnosis. Participant 111: 39, White, Midwest, private insurance, self-employed, some college, married, >100K; received consultation only in 2020.

Some participants reported that their FCC clinicians were able to find a balance, as one participant described: “They were honest, yet presented challenging information with compassion and hope” (Participant 256: 27, White, Midwest, private insurance, employed full-time, doctorate degree, married, >100K; received neonatal intervention and perinatal palliative care in 2021).

Participants with positive comments about compassion (*n* = 11) had a mean total PCC-FCC score of 107 (SD = 6), compared to those with negative comments (*n* = 13), whose mean total score was 71 (SD = 15) (Table 4).

Belonging: Belonging was rooted in a patient’s profound sense of acceptance, value, and deep connection to the healthcare team. FCCs were central to the family narratives of patients during one of the most challenging and impactful periods of their lives. In addition, many patients traveled long distances or relocated entirely to access FCC care, highlighting the significant commitment involved and the need for a sense of unity and connection beyond meeting their medical needs. Many participants fondly recalled the meaningful friendships they built with their FCC teams, recounting how they were embraced “like family”. They described a genuine feeling of FCC team members becoming an integral part of their own families as well, as they sought to form a new family identity that reframed the fetal condition as part of their unique story. As not everyone has the means to travel long distances or relocate entirely for care, this highlights a potential disparity in access to FCCs and this level of PCC for those who do not have the same resources. One participant demonstrated this by saying “They knew me, my husband, and our baby. I was so thankful that the facility was available. We had to travel a couple hours and relocate for delivery, but I wouldn’t want it any other way” (Participant 136: 33, White, Midwest; private insurance, employed full-time, bachelor’s degree, married, >100K; received expectant management in 2019).

The FCC model demonstrated both strengths and limitations in fostering a sense of belonging. On the one hand, some participants felt that the collaborative nature of large teams created a supportive environment akin to a close-knit family. However, the numerous team members sometimes resulted in a lack of provider consistency, making it difficult to establish enduring relationships and predict interactions with each member. Additionally, although overseeing patient care throughout the prenatal journey and coordinating neonatal care planning can promote a sense of enduring unity, the conclusion of FCC post-pregnancy may disrupt this continuity, potentially affecting the patient’s sense of belonging.

I understand the notion of seeing many providers for care, but I felt like I did not have a great/trusting relationship with any one provider, which I did in my past high-risk pregnancies. Because of that, I felt like my end result was undesirable. I ended up in the hospital and induced really early but felt as if some of the providers believed my issues were real, while others treated me as if I were making up the issue. After my baby was born, I felt alone and uncared for by the medical team. Participant 194: 34, Black or African American, Northeast, private insurance, homemaker, bachelor’s degree, married, 50K–74K; received fetal intervention and neonatal intervention in 2019.

To cultivate a sense of belonging, FCC teams engaged in ongoing processes that expanded upon the foundational themes of trust, clarity, comprehensive care, and compassion. The intersection of these themes is evident in the quote below, where they come together to shape an environment of care that, even in the face of heartbreaking outcomes, resulted in a positive and meaningful care experience:

I loved the care team meeting. My husband and myself had a meeting with every provider that would take part in our son’s care: cardio, neuro, neonatologist, NICU nurse, palliative. It was amazing to hear each specialty speak and formulate a plan together. The compassion in that room was palpable. We knew step by step what we could expect and what would happen if something changed. It was the best experience I could have hoped for in a very stressful time. In the end, we chose to deliver at our local hospital where we chose hospice for our son. A week later we received a sympathy card from our fetal care team signed by all the physicians, coordinators, and nurses. I loved our experience. Participant 133: 31, White, Midwest, private insurance, employed full-time, associate degree, married, 75K–99K; received perinatal palliative care in 2020.

Participants with positive comments about belonging (*n* = 11) had a mean total PCC-FCC score of 110 (SD = 2), compared to those with negative comments (*n* = 8), whose mean total score was 75 (SD = 13) (Table 4).

## 4. Discussion

This study provides new and valuable insights into PCC experiences in FCCs, highlighting areas of strength and opportunities for improvement, with a focus on potential sociodemographic disparities. Our study found high overall PCC scores, which align with previous research indicating that coordinated care improves satisfaction with perinatal care [13]. However, the variation in PCC-FCC scores and participant comments reflects differences in experiences, as elucidated by the qualitative analysis and mirrored by the quantitative findings. This suggests that not all patients experience PCC equally.

The higher PCC-FCC scores among participants with public insurance highlight an area marked by limited and conflicting findings. Research comparing Medicaid and private insurance in terms of patient satisfaction has generally overlooked pregnant patients, particularly those at high risk, and the results among other populations have been mixed. A 2019 meta-analysis of 34 studies found that Medicaid insurance was associated with lower success rates in scheduling primary care and specialty appointments compared to private insurance [14]. In contrast, a 2021 study of 149,290 individuals in 18 states found that those with private insurance reported poorer access to care, higher costs, and lower satisfaction compared to those covered by publicly sponsored programs [15]. FCC insurance issues can be particularly unique. Variable coverage for maternal–fetal surgery procedures often necessitates detailed discussions between clinicians and insurance representatives to explain the complexities and justify deviations from standard policies [16]. These discussions are typically handled on a case-by-case basis, consuming a significant portion of an FCC clinical coordinator’s time. Given that less than 20% of our sample had public insurance, it is plausible that the barriers faced by these patients occur primarily before they reach the FCC. Thus, those who do gain access may perceive the coordinated multidisciplinary specialist care as highly person-centered compared to the care that they received prior to reaching the FCC, a finding consistent with previous research examining FCC patient journeys [7]. Importantly, the association between insurance type and PCC-FCC scores was not replicated in the PPCR scores, suggesting that our scale may be particularly sensitive to capturing experiences tied to insurance or another similar-but-separate construct.

The qualitative themes of trust, clarity, comprehensive care, and compassion align with key components of PCC identified in the literature, offering unique perspectives on these concepts within FCCs [4,17,18]. The theme of belonging, however, provides a distinct perspective underscored by evidence from medical anthropology, social sciences, and humanities, emphasizing the links between belonging, health, and healthcare [19]. Although primarily centered on immigrant populations, this evidence offers insights applicable to marginalized individuals and those facing existential displacement, such as those transitioning from anticipating a normal pregnancy to a high-risk one [20]. These individuals encounter political and social non-belonging, particularly within the current reproductive health policy landscape, where restrictive abortion laws and societal biases against termination and disability intersect [21]. Individuals facing severe fetal anomalies also experience a loss of basic social connections and norms, struggling to participate in traditional pregnancy rituals and feeling disconnected from typical pregnancy environments, where images of happy and healthy mothers and babies fail to reflect their own feelings and experiences [21,22]. Therefore, FCCs play a vital role in fostering a sense of belonging, offering patients comfort and understanding from clinical teams, regardless of their chosen care pathway [6,7,9]. Additionally, FCCs facilitate connections to new and supportive communities like fellow NICU parents or condition-specific support groups. Given the importance of belonging in fostering psychological resilience, it is evident that this aspect of PCC holds unique significance in FCC settings [23,24,25]. However, the ability of some individuals to access and maintain care at FCCs may be hindered by sociodemographic barriers, ultimately affecting their sense of belonging. For example, many participants in this pilot described needing to drive for hours or relocate entirely for care, and 25% of participants reported facing financial challenges related to FCC care.

This study must be understood in the context of the following limitations: The significant lack of diversity in the sociodemographic characteristics of our pilot sample limited the analysis of disparities in areas such as race and ethnicity, thus limiting the generalizability of our findings. Similarly, self-selection bias may have influenced the characteristics of our sample, and their experiences may not represent the entire population. In addition, geographic data were collected at the regional level, disabling site- or even state-specific analysis.

The evidence from this study provides useful insight for clinicians into patients’ experiences and practical context when using the validated scale. Additionally, there are several indications for future research. First, the joint display highlights that some themes, such as belonging, may lack appropriate corresponding PCC-FCC Scale items, and certain items may not fully capture the unique PCC themes. For example, items specifically addressing how FCC teams managed uncertainty might be missing, and some participants’ quotes seem misaligned with their scores on corresponding items. This discrepancy may arise from question phrasing, such as emphasizing whether the FCC team cared about them versus provided specific services or information. This distinction could further differentiate the two scale factors (cared for vs. cared about). Planned national-level mixed-methods assessments of the scale will allow for continued refinement of the concept and measurement of PCC in FCCs with more diverse samples [1]. Authors should discuss the results and how they can be interpreted from the perspective of previous studies and of the working hypotheses. The findings and their implications should be discussed in the broadest context possible. Future research directions may also be highlighted.

## 5. Conclusions

The findings of this mixed-methods pilot study provide a comprehensive understanding of PCC experiences in FCCs, highlighting both strengths and areas for improvement. High overall PCC scores and positive comments indicate effective care coordination, respectful care, and patient education, although variability in patient experiences suggests inconsistencies in areas of expectation setting, preparation for post-intervention maternal health, and offering psychosocial support. Participants with public insurance reported higher PCC, a finding that warrants further investigation given the mixed results in the literature and highlights the need for analyses of disparities to be conducted with more diverse samples. The identified themes—trust, clarity, comprehensive care, compassion, and belonging—underscore the need for FCCs to continuously address both the medical and emotional needs of patients facing fetal conditions.

## Figures and Tables

**Table 1:** Sociodemographic data of included and excluded participants in the analysis (total *N* = 258/included *n* = 247/excluded *n* = 11).

Characteristics of Total Number of Participants	Included Participants—*n* (%)	Excluded Participants—*n* (%)
Age	Range 19–43, Mean = 31 ± 4.5	Range 24–39, Mean = 30.7 ± 4.4
Gender by pronouns		
She/her/hers	242 (97.9)	11 (100)
They/them/theirs	3 (1)	0 (0)
He/him/his	1 (0.4)	0 (0)
Other	1 (0.4)	0 (0)
Race and ethnicity *		
Non-Hispanic White	221 (89.4)	10 (90.1)
Hispanic or Latino	5 (1.9)	0 (0)
Hispanic or Latino, White	5 (1.9)	0 (0)
Asian	4 (1.6)	0 (0)
Black or African-American	4 (1.6)	0 (0)
Black or African-American, White	4 (1.6)	0 (0)
Asian, White	3 (1.1)	1 (9.1)
Other	1 (0.4)	0 (0)
Relationship status		
Married	216 (87.4)	8 (72.7)
In a relationship, not married	28 (11.3)	2 (18.2)
Single	3 (1.2)	1 (9.1)
Insurance type		
Private	202 (81.7)	5 (45.5)
Public	43 (17.4)	5 (45.5)
No insurance	2 (0.8)	1 (9.1)
Employment status		
Full-time	169 (68.4)	6 (54.5)
Homemaker	46 (18.6)	3 (27.3)
Part-time	17 (6.8)	1 (9.1)
Unemployed	13 (5.2)	1 (9.1)
Student	2 (0.8)	0 (0)
Combined household income		
USD > 100,000	98 (39.7)	6 (54.5)
USD 50,000–99,999	95 (38.5)	1 (9.1)
USD 20,000–49,000	42 (17)	4 (36.4)
USD < 20,000	11 (4.5)	0 (0)
No answer	1 (0.4)	0 (0)
I faced financial challenges related to my care		
No	183 (74.1)	0 (0)
Yes	64 (25.9)	1 (9)
No answer	0 (0)	10 (91)
Education level		
Bachelor’s degree	88 (35.6)	4 (36.4)
Graduate or postgraduate degree	72 (29.1)	2 (18.2)
Some college or associate degree	50 (20.2)	4 (36.4)
High school graduate or equivalent	23 (9.3)	1 (9.1)
Trade/technical/vocational training	11 (4.5)	0 (0)
Some high school, no diploma	3 (1.2)	0 (0)
Primary language		
English	244 (98.8)	11 (100)
Other	3 (1.2)	0 (0)
I felt discriminated against during my care		
No	235 (95.1)	8 (72.7)
Yes	12 (5)	2 (18.2)
No answer	0 (0)	1 (9.1)
Region in which care was received		
Midwest	152 (61.5)	4 (36.4)
Northeast	37 (14.9)	3 (27.3)
West	24 (9.7)	1 (9.1)
Southeast	20 (8.0)	2 (18.2)
Southwest	14 (5.6)	1 (9.1)
Year care was received		
2020	70 (28.3)	2 (18.2)
2021	61 (24.4)	3 (27.3)
2018	47 (18.6)	0 (0)
2019	46 (18.6)	3 (27.3)
2017	23 (10.1)	3 (27.3)
Type of care received		
Fetal intervention	72 (28.3)	1 (9.1)
Fetal intervention + neonatal intervention	49 (19)	0 (0)
Expectant management (no intervention)	40 (17.1)	4 (36.4)
Neonatal intervention	28 (12)	2 (18.2)
Perinatal palliative care (expected loss)	20 (8.5)	1 (9.1)
Consultation only	10 (3.9)	0 (0)
Fetal intervention + neonatal intervention + palliative care	9 (3.9)	0 (0)
Fetal intervention + palliative care	7 (2.7)	2 (18.2)
Neonatal intervention + palliative care	5 (1.9)	0 (0)
Termination of pregnancy	5 (1.9)	1 (9.1)
Fetal intervention + termination of pregnancy	2 (0.8)	0 (0)

* Participants were able to choose more than 1 race/ethnicity type.

**Table 2:** Summary of item scoring from highest to lowest mean.

Item	Mean (SD)	Median [IQR]
F1: Someone from the fetal care center explained the reasons for the care that I received.	3.81 (0.48)	4.00 (4.00, 4.00)
F1: The fetal care center team explained things in a way that I could understand.	3.76 (0.52)	4.00 (4.00, 4.00)
F1: The fetal care center environment supported my physical needs.	3.74 (0.55)	4.00 (4.00, 4.00)
F1: The fetal care center team used words that was respectful of my pregnancy or baby/babies.	3.76 (0.57)	4.00 (4.00, 4.00)
F1: The fetal care center team answered my questions.	3.72 (0.57)	4.00 (4.00, 4.00)
F1: Clinical care at the fetal care center was done with compassion.	3.74 (0.60)	4.00 (4.00, 4.00)
F1: The fetal care center team listened to me.	3.71 (0.58)	4.00 (4.00, 4.00)
F1: The fetal care center team checked that I understood information that was given to me.	3.69 (0.59)	4.00 (3.00, 4.00)
F1: When possible, the fetal care center team gave me the time I needed to make decisions.	3.65 (0.58)	4.00 (3.00, 4.00)
F1: The fetal care center team cared about my health as well as the health of my fetus/baby.	3.69 (0.66)	4.00 (4.00, 4.00)
F1: My visits with the fetal care center team were well coordinated.	3.65 (0.63)	4.00 (3.00, 4.00)
F2: I had a member of the fetal care center team I could contact when I had a question or problem.	3.64 (0.68)	4.00 (3.00, 4.00)
F1: At the fetal care center, I was told about all the possible options for my pregnancy.	3.60 (0.69)	4.00 (3.00, 4.00)
F1: Members of the fetal care center team told me about the options for my pregnancy in a compassionate way.	3.59 (0.69)	4.00 (3.00, 4.00)
F1: The fetal care center team helped me make decisions that were best for me and my family.	3.59 (0.69)	4.00 (3.00, 4.00)
F1: During my visit, the fetal care center team took time to learn about me and my story.	3.58 (0.69)	4.00 (3.00, 4.00)
F1: The fetal care center team followed through on my plan of care.	3.59 (0.70)	4.00 (3.00, 4.00)
F1: I felt supported in my decisions by the fetal care center team.	3.60 (0.73)	4.00 (3.00, 4.00)
F1: Reverse coded: I felt pressured into a decision by the fetal care center team.	3.57 (0.78)	4.00 (3.00, 4.00)
F2: The fetal care center team prepared me for changes in my care.	3.43 (0.78)	4.00 (3.00, 4.00)
F1: Reverse coded: I felt like I had to fight for my needs and wishes	3.44 (0.92)	4.00 (3.00, 4.00)
F2: The fetal care center team gave me resources to help me learn about the condition(s) diagnosed.	3.33 (0.86)	4.00 (3.00, 4.00)
F2: The counseling from the fetal care center prepared me well for my care experience.	3.29 (0.87)	4.00 (3.00, 4.00)
F2: I felt satisfied with the amount of interaction I had with the fetal care center team after I left their care.	3.33 (0.91)	4.00 (3.00, 4.00)
F2: The fetal care center team asked me about emotional health concerns.	3.24 (0.89)	4.00 (3.00, 4.00)
F2: The fetal care center team asked me about my expectations and hopes throughout my care.	3.20 (0.87)	3.00 (3.00, 4.00)
F2: The fetal care center team gave me resources to help with my emotional health.	3.18 (0.90)	3.00 (3.00, 4.00)
F2: The fetal care center team helped me learn how to talk to family and friends about my pregnancy.	2.83 (1.00)	3.00 (2.00, 4.00)

F1 = Factor 1: reflects the sense of being cared *for* through respectful and compassionate caregiving tasks, such as technical competence and effective communication. F2 = Factor 2: signifies feeling cared *about* by addressing health-related quality-of-life needs, including genuine provider connections and meeting psychosocial needs.

**Table 3:** PCC-FCC and PPPC-R scores by sociodemographic groups.

Characteristics	PCC-FCC Total Mean (SD)	*p*-Value	PCC-FCC Factor 1 Mean (SD)	*p*-Value	PCC-FCC Factor 2 Mean (SD)	*p*-Value	PPPC-R Total Mean (SD)	*p*-Value
Household Income								
USD < 100,000 (*n* = 148)	96 (17)	0.302	68 (11)	0.458	28 (7)	0.307	3.59 (0.63)	0.590
USD > 100,000 (*n* = 98)	97 (14)		69 (9)		28 (6)		3.64 (0.49)	
Insurance								
Private (*n* = 203)	96 (16)	0.011 *	68 (10)	0.088	28 (6)	0.008 *	3.61 (0.56)	0.235
Public insurance (*n* = 42)	100.45 (17)		70 (11)		31 (7)		3.63 (0.66)	
Education								
Some high school, high school graduate or equivalent, trade/technical/vocational training	95 (22)	0.584	66 (15)	0.473	29 (8)	0.489	3.46 (0.82)	0.937
Bachelor’s or associate degree	96 (15)		68 (10)		28 (7)		3.62 (0.53)	
Graduate or postgraduate degree	98 (14)		28 (7)		29 (6)		3.66 (0.51)	

* All differences statistically significant at a *p* < 0.05 level.

**Table 4:** Joint display: qualitative data and convergent quantitative data.

Sample Quotes by Theme: Subtheme and Positive vs. Negative Sentiment	Quoted Participant’s Scores on Related Items	Mean (SD) Total Score by Theme Sentiment
**Trust**: providing PPC amid uncertainty		
(+) “Fetal care is so dynamic and, in my case, even knowing what the care plan was, there still was so many unknowns. I never once doubted what the fetal care team was telling me or how they were advising me. Was it hard to hear? Absolutely. But they are the reason *I have two happy and thriving babies today*.”—Participant 211: 33, White, West, private insurance, employed full-time, bachelor’s degree, married, >100K. Received fetal intervention in 2021	The team prepared me for changes in my care: 3 (agree)	(+) (*n* = 7) 103 (7)
(—) “I feel that we should have been told about the hospital that specializes in my daughter’s specific defects and given the option to explore that second opinion.”—Participant 95: 26, White, Midwest, public insurance, homemaker, some college, married, 35K–49K. Received expectant management in 2020	The team helped me make decisions that were best for me and my family: 2 (disagree) * The team asked me about my expectations and hopes throughout my care: 1 (disagree strongly) * At the fetal care center, I was told about all the possible options for my pregnancy: 3 (agree)	(—) (*n* = 15) 75 (20)
**Clarity**: communicating all possible outcomes		
(+) “We knew step by step what we could expect and what would happen if something changed.”—Participant 133: Age 31, White, Midwest, private insurance, employed full-time, associate degree, married, 75K–99K. Received perinatal palliative care in 2020	The counseling from the fetal care center prepared me well for my care experience: 4 (strongly agree)	(+) (*n* = 6) 106 (5)
(—) “Our son was removed from support in the NICU when he was four days old. My care plan was really surrounded around the assumption that he would be very sick during his NICU stay but would ultimately leave the hospital as a medically complex kiddo. I almost wish I would have received aspects of care geared towards those looking at palliative care as well so that I would have been more prepared when faced with that outcome. At the time, I don’t think I wanted to even consider it as an option, so I understand how difficult it must be to provide the appropriate care for each patient and family.”—Participant 210: 32, White, West, private insurance, employed full-time, bachelor’s degree, married, >100K. Received fetal intervention, neonatal intervention, and perinatal palliative care in 2020	The counseling from the fetal care center prepared me well for my care experience: 3 (agree) The fetal care center team prepared me for changes in my care: 4 (strongly agree)	(—) (*n* =17) 80 (18)
**Comprehensive Care**: coordination, continuity, and focus on both pregnant person *and* fetus/baby		
(+) “My center coordinated so well with my home hospital where I delivered after fetal surgery. I can still reach out to my nurse coordinator to this day with any questions.”—Participant 166: 32, White, Midwest, private insurance, employed full-time, master’s degree, married, >100K. Received fetal intervention in 2021	My visits were well coordinated: 4 (agree strongly) I felt satisfied with the amount of interaction I had with the FCC team after I left their care: 4 (agree strongly)	(+) (*n* = 11) (107) (5)
(—) “I did not fully understand what I was signing up to happen to my body post-surgery. I was only thinking about the baby, and it was never fully explained to me. It wouldn’t have changed by decision, but I might have been more prepared to face a lot of difficulty.”—Participant 174: 31, White, Midwest, private insurance, homemaker, bachelor’s degree, married, 75K–99K. Received fetal intervention in 2018	The fetal care center team cared about *my health* as well as the health of my fetus/baby: 3 (agree) The counseling from the fetal care center prepared me well for my care experience: 1 (disagree strongly) *	(—) (*n* = 16) 83 (21)
**Compassion**: holistic care, routine with empathy		
(+) “They were very compassionate towards me and my family. Being that I lived 6 h away and struggling to find somewhere to stay while my baby was in NICU—the entire team provided me all resources needed to stay locally until my baby could be discharged.”—Participant 260: 34, Latino, West, public insurance, employed part-time, bachelor’s degree, married, 75K–99K. Received fetal intervention in 2019	During my visit, the fetal care center team took time to learn about me and my story: 4 (agree strongly)	(+) (*n* = 11) 107 (6)
(—) “I will be dealing my entire life with diagnosed PTSD from the whole experience. It wasn’t all bad, just a lot of casually staring horrible things about me and my baby that were extremely worrisome.”—Participant 185: Age 29, White, Southwest, private insurance, employed full-time, master’s degree, married, >100K. Received fetal intervention in 2020	Clinical care at the fetal care center was done with compassion: 2 (disagree) * The fetal care center team asked me about emotional health concerns: 2 (disagree) *	(—) (*n* = 13) 71 (15)
**Belonging**: sharing the load, balancing clinician education with patient connection		
(+) “My fetal care team took a scary, unknown situation that I suddenly found myself in and gave me peace of mind and the knowledge that we would handle this as a team and that I wasn’t alone.”—Participant 161: 29, White, Midwest, private insurance, employed full-time, some college, married, 75K–99K. Received fetal intervention and neonatal intervention in 2021	The team helped me make decisions that were best for me and my family: 4 (strongly agree)	(+) (*n* = 11) 110 (2)
(—) “I didn’t even get the names of the doctor who actually did my 1st surgery because it wasn’t the doctor I was told and the ones who did my C-section I could tell they were fresh students and with my extremely fragile circumstances I don’t think it was appropriate.”—Participant 82: 25, White, Southeast, private insurance, employed full-time, some college, married, 50K–75K. Received fetal intervention, and termination of pregnancy care in 2021	The fetal care center team prepared me for changes in my care: 2 (disagree) * I had a member of the fetal care center team I could contact when I had a question or problem: 2 (disagree) *	(—) (*n* = 8) 75 (13)

* Indicates that the participant’s answer is at least one Likert-scale point lower than the entire sample’s mean score.

## Data Availability

The data that support the findings of this study are not publicly available due to their containing information that could compromise the privacy of the research participants, but they are available from the corresponding author K.F. upon reasonable request.

## References

[1] Wilpers A., White M., Austin M., Bahtiyar M.O., Francis K., Emery S., Wall D., Somers L., Wool C. (2024). Development and Validation of a Scale to Measure Person-Centered Care in Fetal Care Centers. Fetal Diagn. Ther..

[2] Eyerly-Webb S.A., Ylvisaker H., Beh M., Lim F.Y., Liechty K., Velasco P., Dion E., Snowise S., Lillegard J.B., Feltis B. (2022). Understanding the Care Journey and Needs of Advanced Fetal Care Center Patients. Perm. J..

[3] Hansen K., Akerson V. (2023). Practicing Trauma-informed Care with Parents Expecting a Baby Diagnosed with Congenital Anomaly. J. Soc. Work End-of-Life Palliat. Care.

[4] Richardson W.C., Berwick D.M., Bisgard J.C., Bristow L.R., Buck C.R., Cassel C.K. (2001). Crossing the Quality Chasm: A New Health System for the 21st Century.

[5] Ralston S.J., Leuthner S.R., American College of Obstetricians and Gynecologists, Committee on Ethics, American Academy of Pediatrics, Committee on Bioethics (2011). Maternal-fetal intervention and fetal care centers. Pediatrics.

[6] Colicchia L.C., Snowise S., Wunderlich W., Schulte A., Sidebottom A.C. (2022). Maternal decision-making about intrauterine myelomeningocele repair: A qualitative exploration. Am. J. Obstet. Gynecol..

[7] Wilpers A., Goldblatt Hyatt E., Bahtiyar M.O., Hu Y., Leon-Martinez D., Chervenak F.A., McCoyd J. (2023). “We All Want to Be Able to Tell You Something Hopeful”: Clinicians’ Experiences Providing Maternal-Fetal Surgery Counseling. Fetal Diagn. Ther..

[8] Wilpers A., Lynn A.Y., Eichhorn B., Powne A.B., Lagueux M., Batten J., Bahityar M.O., Gross C.P. (2022). Understanding Sociodemographic Disparities in Maternal-Fetal Surgery Study Participation. Fetal Diagn. Ther..

[9] Shao B., Chen J.S., Kozel O.A., Tang O.Y., Amaral-Nieves N., Sastry R.A., Watson-Smity D., Monteagudo J., Luks F.I., Carr S.R. (2022). Postnatal Myelomeningocele Repair in the United States: Rates and Disparities Before and After the Management of Myelomeningocele Study Trial. Neurosurg..

[10] Curry L., Nunez-Smith M. (2014). Mixed Methods in Health Sciences Research: A Practical Primer.

[11] Ryan B.L., Brown J.B., Tremblay P.F., Stewart M. (2019). Measuring Patients’ Perceptions of Health Care Encounters: Examining the Factor Structure of the Revised Patient Perception of Patient-Centeredness (PPPC-R) Questionnaire. J. Patient Cent. Res. Rev..

[12] Marshall C., Rossman G.B. (2014). Designing Qualitative Research.

[13] Kroll-Desrosiers A.R., Crawford S.L., Simas T.A.M., Rosen A.K., Mattocks K.M. (2016). Improving pregnancy outcomes through maternity care coordination: A systematic review. Women’s Health Issues.

[14] Hsiang W.R., Lukasiewicz A., Gentry M., Kim C.Y., Leslie M.P., Pelker R., Forman H.P., Wiznia D.H. (2019). Medicaid patients have greater difficulty scheduling health care appointments compared with private insurance patients: A meta-analysis. INQUIRY J. Health Care Organ. Provis. Financ..

[15] Wray C.M., Khare M., Keyhani S. (2021). Access to care, cost of care, and satisfaction with care among adults with private and public health insurance in the US. JAMA Netw. Open..

[16] Wilpers A., Bahtiyar M.O., Wall D., Kobler K., Sadler L., Dixon J., Kennedy H.P. (2021). Narrative Inquiry into Nursing Care of Pregnant Women and Families in Specialized Fetal Diagnosis and Treatment Settings. J. Obstet. Gynecol. Neonatal Nurs..

[17] De Labrusse C., Ramelet A.S., Humphrey T., Maclennan S.J. (2016). Patient-centered care in maternity services: A critical appraisal and synthesis of the literature. Women’s Health Issues.

[18] Tunçalp Ӧ., Were W.M., MacLennan C., Oladapo O.T., Gülmezoglu A.M., Bahl R., Daelmans B., Mathai M., Say L., Kristensen F. (2015). Quality of care for pregnant women and newborns—The WHO vision. BJOG.

[19] Mattes D., Lang C. (2021). Embodied belonging: In/exclusion, health care, and well-being in a world in motion. Cult. Med. Psychiatry.

[20] Bendixsen S.K.N. (2020). Existential displacement: Health care and embodied un/belonging of irregular migrants in Norway. Cult. Med. Psychiatry.

[21] McCoyd J.L. (2009). What do women want? Experiences and reflections of women after prenatal diagnosis and termination for anomaly. Health Care Women Int..

[22] McCoyd J.L.M., Curran L., Munch S. (2020). They say, “If you don’t relax you’re going to make something bad happen”: Women’s emotion management during medically high-risk pregnancy. Psychol. Women Q..

[23] Hynan M.T., Mounts K.O., Vanderbilt D.L. (2013). Screening parents of high-risk infants for emotional distress: Rationale and recommendations. J. Perinatol..

[24] Kim A.J.H., Servino L., Bircher S., Feist C., Rdesinski R.E., Dukhovny S., Shaffer B.L., Saxton S.N. (2022). Depression and socioeconomic stressors in expectant parents with fetal congenital anomalies. J. Matern.-Fetal Neonatal Med..

[25] Cole J.C.M., Moldenhauer J.S., Berger K., Cary M.S., Smith H., Martino V., Rendon N., Howell L.J. (2016). Identifying expectant parents at risk for psychological distress in response to a confirmed fetal abnormality. Arch. Womens Ment. Health.

